# Commentary: Negative association between Body Roundness Index and bone mineral density: insights from NHANES

**DOI:** 10.3389/fnut.2025.1555355

**Published:** 2025-03-28

**Authors:** Chenglong Wang, Xuelei Zhang

**Affiliations:** ^1^Department of Orthopedics, Taizhou Hospital of Zhejiang Province Affiliated to Wenzhou Medical University, Taizhou, China; ^2^Department of Ultrasound, Taizhou Hospital of Zhejiang Province Affiliated to Wenzhou Medical University, Taizhou, China

**Keywords:** body roundness index, bone mineral density, osteoporosis, obesity, NHANES

## 1 Introduction

We read with great interest the article by Ding et al. (1) entitled “*Negative association between Body Roundness Index and bone mineral density: insights from NHANES*.” In this study, the authors explored the relationship between body roundness index (BRI) and total bone mineral density (BMD) in U.S. adults. BRI, a newer anthropometric measure, has been shown to more comprehensively reflect visceral fat distribution compared to other traditional indices. Unlike other measures, BRI does not include weight in its calculation; instead, it uses waist circumference, height, and a formula to estimate visceral adipose tissue. The authors constructed three weighted multivariate regression models to explore the relationship between BRI and BMD: Model 1 was unadjusted, Model 2 adjusted for key demographic variables, and Model 3 adjusted for all covariates. The key findings demonstrate a notable inverse relationship between BRI and BMD, thus authors concluded that a higher BRI is associated with lower BMD and a potentially greater risk of developing osteoporosis, advocating for the use of BRI as a valuable marker for early intervention. We commend the authors for their rigorous study design and analytical efforts; however, we would like to raise several concerns and offer suggestions for further consideration to help improve their investigations.

## 2 Adjustment for body mass index

In Model 3, the authors commendably adjusted for a comprehensive set of covariates, including age, gender, race, education level, poverty-to-income ratio, body mass index (BMI), smoking status, alcohol consumption, activity level, high-density lipoprotein cholesterol, total cholesterol, fasting plasma glucose, triglycerides, vitamin D3, phosphorus, total calcium, creatinine, alanine aminotransferase, diabetes status, and arthritis status. However, the inclusion of BMI as a covariate introduces potential multicollinearity due to the overlapping components in BMI and BRI calculations.

As indicated in Table 1 of the original study, there was a significant positive correlation between BRI and BMI, while BMD levels did not vary substantially across different BRI tertiles. Notably, in Models 1 and 2, BRI was consistently positively associated with BMD, but in Model 3, the association was reversed and became negative. This reversal is likely due to the adjustment for BMI, as a recent study found that BRI negatively associated with the prevalence of osteoporosis in a weighted multivariate logistic regression analysis that did not adjust for BMI (2). Notably, collinearity among the variables can lead to inflated standard errors, reversed effects, or non-significant effects. The observed reversal in Model 3 without a notable increase in standard deviations suggests the need for a variance inflation factor analysis. This analysis would help the authors to rule out collinearity between BRI and BMI and clarify why the results of Model 3 differ from those generated by Models 1 and 2. These two studies have reached completely opposite conclusions, which prompted us to reconsider whether BMI adjustment is necessary when investigating the relationship between new anthropometric measures and diseases in the future.

## 3 Visceral adiposity and osteoporosis

In fact, through direct dual energy X-ray absorptiometry measurements, visceral adiposity is negatively associated with BMD (3–5). Visceral fat is more metabolically active than other types of fat, secreting a variety of cytokines, including inflammatory ones that disrupt bone remodeling processes. The protective role of mechanical load may be diminished by the adjustment of BMI, which could highlight the adverse effects of inflammation on bones. We commend the authors for their detailed discussion of the potential mechanisms through which visceral fat accumulation promotes osteoporosis.

## 4 Diagnostic ability

The authors suggest using the BRI as a valuable marker for clinically assessing the risk of osteoporosis and for achieving early intervention. However, the diagnostic ability of BRI was not evaluated in the study. We encourage the authors to perform receiver operating characteristic curve analysis to compare the diagnostic ability of BRI with traditional obesity assessment indicators (not limited to BMI) and other novel measures, such as the Weight-adjusted Waist Index (6), A Body Shape Index (7), Abdominal Volume Index (8), Visceral Adiposity Index (9), and Lipid Accumulation Product index (10). Beyond the receiver operating characteristic analysis, incorporating additional metrics such as the net reclassification index would help establish whether BRI provides incremental diagnostic value over traditional measures, thereby highlighting the significance of this study.

## 5 Discussion

This study provides an innovative exploration of the relationship between BRI and BMD in U.S. adults; however, we believe that the findings should be interpreted with caution. Specifically, we emphasize that when investigating the association between novel anthropometric measures and osteoporosis, it is crucial to consider the potential collinearity with BMI. In addition, longitudinal studies that examine the impact of weight loss or redistribution of body fat on BMD across BRI tertiles would provide valuable insights into causal relationships. We hope that our insights will assist readers in interpreting the findings more effectively and offer additional perspectives for future research.
